# Steal Syndrome in Free Flap Microvascular Reconstruction of the Lower Extremity: Systematic Review of Incidence, Risk Factors, and Surgical Management

**DOI:** 10.3390/bioengineering12060647

**Published:** 2025-06-12

**Authors:** Georgios Karamitros, Ilias Iliadis, Raymond A. Pensy, Gregory A. Lamaris

**Affiliations:** 1 Division of Plastic and Reconstructive Surgery, R. Adams Cowley Shock Trauma Center, University of Maryland Medical Center, Baltimore, MD 21201, USA; rpensy@som.umaryland.edu (R.A.P.); glamaris@som.umaryland.edu (G.A.L.); 2 Medical School, University of Ioannina, 45110 Ioannina, Greece; ilias.s.iliadis@gmail.com

**Keywords:** steal syndrome, free flap, microvascular reconstruction, peripheral vascular disease, diabetes, amputation, flap revision, anastomosis technique, vascular pedicle, myocutaneous flap, anastomotic revision, post-operative complications, ischemia, blood flow, flap survival, recipient artery, vascular complications, clinical predictors, anatomical predictors, surgical outcomes, reconstructive surgery, flap ischemia, vascular health, ischemic necrosis, limb salvage surgery

## Abstract

**Background**: Steal syndrome in the setting of microvascular reconstruction refers to a phenomenon whereby blood flow is diverted from the native tissue to the free flap, leading to ischemia and potential limb loss. In the present study, we aim to comprehensively evaluate the occurrence and management of steal syndrome in free flap reconstruction of the lower extremities. **Methods**: A thorough literature search was conducted across the MEDLINE, Embase, Cochrane Library, and Scopus databases up to 29 January 2025. Studies were selected based on predefined inclusion criteria focusing on free flap microvascular reconstruction in the lower extremities with a focus on steal syndrome. Two independent reviewers assessed and extracted data. **Results**: Three studies were included, involving seven patients, with a mean age of 65.66 ± 5.89 years, who developed steal syndrome following free flap microvascular reconstruction. The most common revision involved below-the-knee amputation (BKA) due to ischemic complications. Comorbidities such as peripheral vascular disease (PVD), diabetes, and hypertension were present in all cases. The majority of anastomoses (85.7%) were end-to-side (ETS), with only one case utilizing a flow-through configuration. The majority of cases (n = 5, 71.4%) were reconstructed using latissimus dorsi (LD) flaps, with the remaining two cases using rectus abdominis (n = 1) and gracilis (n = 1) flaps. The recipient vessel was the anterior tibial artery in two patients (28.6%), the dorsalis pedis artery in two patients (28.6%), and the popliteal artery in three patients (42.9%). The most common salvage procedure was below-the-knee amputation (BKA), performed in four patients (57.1%). One patient required revision of the venous anastomosis and flap debridement, followed by a Chopart amputation (n = 1, 14.3%). **Conclusions**: The occurrence of steal syndrome in free flap microvascular reconstruction of the lower extremities is rare but can lead to significant complications, including amputation. The findings indicate that steal syndrome is more likely in patients with pre-existing vascular conditions such as PVD and diabetes. While surgical technique and flap type may influence its development, further studies are needed to identify specific anatomical and clinical predictors. The absence of a unified treatment guideline underscores the need for further investigation into effective management strategies to prevent amputation and optimize patient outcomes.

## 1. Introduction

The primary objectives of reconstruction in complex lower limb injuries are the restoration and preservation of function [1,2]. The reconstruction of lower extremity defects presents a unique challenge due to the limited availability of local soft tissue and vascular supply [3,4,5]. Current reconstructive modalities encompass direct closure, skin grafting, and a variety of flap-based techniques, including muscle flaps, cross-leg flaps, and free flaps [6,7]. Free tissue transfer offers several advantages, including robust vascularity, the ability to provide tissue with similar characteristics to the defect site, minimal size constraints, and superior aesthetic outcomes [8,9,10,11,12,13]. However, free flap reconstruction requires microsurgical expertise, depends on adequate recipient-site perfusion, and is associated with prolonged operative time [14].

R. Adams Cowley Shock Trauma Center, one of the largest Level I trauma hospitals in the United States, has been at the forefront of limb salvage efforts [15]. Here, multidisciplinary collaboration between Orthopedic and Plastic Surgery teams is instrumental in managing complex lower extremity reconstructions, including those necessitated by traumatic injuries, diabetic foot complications, and oncologic resections. One critical consideration in free flap reconstruction is the potential development of steal syndrome—a phenomenon in which blood flow is preferentially diverted away from the native circulation toward the free flap, leading to ischemia in the original vascular territory [16]. Despite its clinical relevance, steal syndrome remains underrecognized and poorly characterized in the context of microvascular reconstruction of the lower extremity.

This systematic review aims to comprehensively evaluate the existing literature on steal syndrome in lower limb reconstruction following microsurgical free flap transfer. By consolidating all reported cases, this study seeks to identify key predictors associated with the development of steal syndrome, as well as patient-specific clinical and anatomical characteristics that may predispose individuals to this vascular complication. Additionally, the review explores potential treatment strategies and serves as a valuable resource for microsurgeons by providing evidence-based insights to optimize patient outcomes and effectively address steal syndrome in lower limb free flap reconstruction.

## 2. Materials and Methods

### 2.1. Guidelines and Ethics

This review was conducted following the protocol registered with the International Prospective Register of Systematic Reviews (PROSPERO) (ID: CRD42025639376) [17]. The study adhered to the Preferred Reporting Items for Systematic Reviews and Meta-Analyses (PRISMA) guidelines to ensure transparency and methodological rigor [18]. Since this review analyzed data already available in the published literature, institutional review board (IRB) approval was not required. Ethical considerations were upheld by exclusively utilizing publicly accessible information, ensuring compliance with established research standards.

### 2.2. Literature Search

Comprehensive systematic literature searches were conducted across multiple databases, including MEDLINE (Ovid), EMBASE (Elsevier), Cochrane Library, and Scopus, up to 29 January 2025. No restrictions were placed on publication dates to ensure a thorough inclusion of all relevant studies. The search strategy was developed in collaboration with a medical librarian and employed a combination of controlled vocabulary and keyword searches using Boolean operators to maximize the scope of the review.

Search terms included variations of the following:

- Terms related to free flap microvascular reconstruction: “Free Tissue Flaps,” “Microvascular Surgery,” “Microanastomosis,” “ Microvascular Reconstruction,” “Free Flap,” and “Free Tissue Graft.”
- Terms related to steal syndrome: “Steal Phenomenon,” “Steal Syndrome,” “Arterial Steal,” “Vascular Steal,” and “Subclavian Steal Syndrome.”
- Search operators were applied to refine results, such as steal NEXT/1 (syndrome* OR phenomenon*)) OR ((arter* OR vascular OR Subclavian) NEXT/1 steal).

The search strategy details, including Boolean operators and database-specific queries, are available in the Supplementary Materials to ensure full transparency and reproducibility.

### 2.3. Study Selection

The systematic review included studies based on predefined inclusion and exclusion criteria structured around the **PICOS** framework (*Population*, *Intervention*, *Comparison*, *Outcomes*, and *Study design*) [19]. The inclusion criteria are as follows:

- Clinical studies investigating the presence of steal syndrome in free flap microvascular reconstruction of the lower extremities.
- Studies reporting quantitative or qualitative data on steal syndrome, including predictors, implicated flaps, and recipient vessels.
- Prospective, retrospective, observational studies, case series, and case reports providing relevant data.
- Studies published in English.

Exclusion Criteria:

- Animal studies or in vitro research.
- Studies focusing on non-free flaps (e.g., pedicled flaps), or reporting the occurrence of steal syndrome unrelated to free flap surgery.
- Studies without clinical relevance or those failing to report outcomes related to steal syndrome.
- Non-English language studies without available translations.

The selection process was conducted by two independent reviewers, who systematically screened the titles and abstracts of all identified studies for relevance. Full texts were assessed for articles with insufficient detail in their abstracts. To streamline the review process, study management and screening were performed using the Covidence platform (https://www.covidence.org/). Any discrepancies between the reviewers were resolved through consultation with a third researcher.

**Figure 1:**
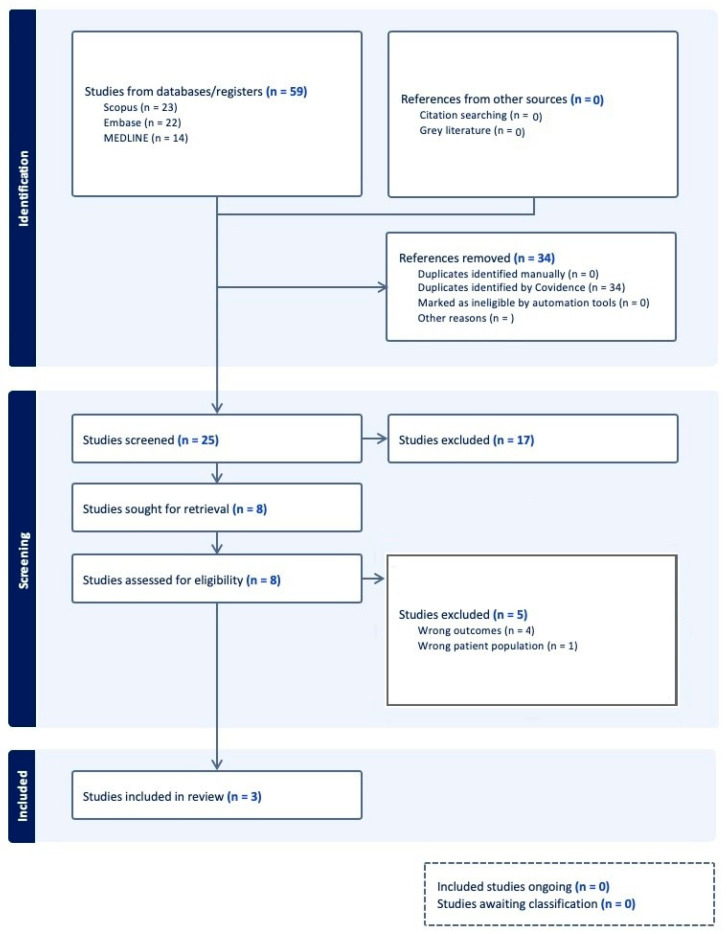
PRISMA flowchart for article selection.

The initial search retrieved 59 articles, which underwent duplicate removal in Covidence, yielding 25 unique studies for further evaluation (Figure 1). These articles were independently reviewed by two authors (G.K. and I.I.), with full-text assessments conducted to determine their relevance to steal syndrome in lower extremity free flap reconstruction. Disagreements regarding study inclusion were adjudicated through discussion with the senior author (G.A.L.).

## 3. Quality Assessment and Risk of Bias

The methodological quality of the included studies was evaluated using validated tools appropriate to each study design. The risk of bias was independently assessed by two reviewers (G.K. and I.I.). For case reports and case series, the Methodological Quality and Synthesis Tool for Case Series and Case Reports was applied, which evaluates eight key domains spanning selection, ascertainment, causality, and reporting [20]. No randomized controlled trials or non-randomized studies were included in this review. Additionally, all articles were graded according to the American Society of Plastic Surgeons’ levels of evidence and grading recommendations to ensure a standardized appraisal of study quality [21].

### Statistical Analysis

Statistical analyses were performed using Microsoft Excel to calculate frequencies, percentages, means, and standard deviations (SDs) for clinical and demographic variables. Categorical variables, such as study design, microvascular reconstruction configuration, complications, and surgical outcomes, were summarized accordingly. Continuous variables, including patient age and sample size, were described using means and SD, assuming a normal distribution of the data. Percentages were calculated by dividing the frequency of each category by the total number of patients in the included studies. Due to the limited number of studies and small sample size, further statistical analysis, including pooled meta-analyses, could not be performed.

## 4. Results

### 4.1. Study Selection

A comprehensive search identified a total of 59 studies across multiple databases, including MEDLINE (Ovid) (14), EMBASE (Elsevier) (22), Cochrane Library (0), and Scopus (23), with the final search executed on 29 January 2025. Following de-duplication in Covidence, 25 unique studies remained for screening.

Following the screening process, studies deemed irrelevant to steal syndrome in lower extremity free flap reconstruction were excluded. After full-text review, further exclusions were made due to lack of methodological rigor, incomplete data, or irrelevance to the study objective. Ultimately, 3 studies were included in the systematic review. The selection process, including reasons for exclusion at various stages, is detailed in the PRISMA flow diagram (Figure 1).

### 4.2. Study Characteristics

A total of three studies were included in the review, with retrospective case series being the most common study design, accounting for 66.6% (n = 2) of the studies. Case reports comprised 33.4% (n = 1) of the studies. Geographically, the majority of the studies (66.6%, n = 2) were conducted in the United States of America, while the remaining study (n = 1) was conducted in Austria. The publication years of the included studies ranged from 1999 to 2003. Table 1 provides an overview of the characteristics of the included studies.

### 4.3. Patient Demographics

The studies included a total of seven patients, all of whom developed steal syndrome and were subjected to amputation, including below-the-knee amputation (BKA) and Chopart amputation. The mean age of the patients was 65.66 ± 5.89 years, with ages ranging from 36 to 73 years across the studies. In terms of sex distribution, the majority of participants were female, with 4 females (57.1%) compared to 3 males (42.9%) (Table 2).

Comorbidities reported across the studies included hypertension (n = 7), diabetes (n = 7), peripheral vascular disease (n = 7), and peripheral neuropathy (n = 1). Active smoking was not reported in any of the patients, and smoking history was not mentioned either. All studies (100%, n = 3) were conducted in tertiary care centers, with two of the studies (66.6%) performed in the same hospital, Lehigh Valley Hospital, and in the same year, 1995 (Table 2).

### 4.4. Clinical Characteristics

All patients were subjected to free flap microvascular reconstruction due to non-healing ulcers of the foot, with two cases involving more extensive soft tissue defects, one of which had necrosis of the fifth toe, with the other having exposure of the Achilles tendon. There were no cases of traumatic reconstruction or limb salvage procedures, and all cases were elective, not reconstructed in the emergency setting. The majority of cases (n = 5, 71.4%) were reconstructed using a latissimus dorsi (LD) flap, while the remaining two cases were reconstructed using rectus abdominis and gracilis flaps. The anterior tibial artery was used as the recipient vessel in two (n = 2, 28.6%), the dorsalis pedis artery was used in 2 cases (n = 2, 28.6%), while the popliteal artery was utilized in three cases (n = 3, 42.9%) (Table 2).

### 4.5. Microvascular Reconstruction Characteristics

In the first study by Sonntag et al. [22], all patients underwent end-to-side anastomoses. In the first case, a reverse saphenous vein graft was used between the latissimus dorsi (LD) flap and the popliteal artery. In the second case, continuity between the deep femoral and popliteal arteries was achieved using a PTFE graft, and a saphenous vein graft was anastomosed between the PTFE graft and the flap pedicle (inferior epigastric artery). In the third case, the thoracodorsal artery of the LD flap was anastomosed to a saphenous vein graft, establishing continuity between the popliteal and dorsalis pedis arteries. In all cases, a single vein anastomosis was performed, utilizing the popliteal vein (Table 3).

In the study by Musser et al. [23], a cephalic vein graft was anastomosed end-to-side to a saphenous vein graft, achieving continuity between the superficial femoral and proximal anterior tibial arteries. In this case, a single vein outflow was used, utilizing the posterior tibial vein.

In the study by Rainer et al. [24], one patient was reconstructed with a flow-through LD flap at the anterior tibial artery, without the use of any vein grafts, and a single vein outflow to the anterior tibial vein. The other two cases presented by Rainer et al. were reconstructed with gracilis and LD flaps, utilizing end-to-side anastomoses without vein grafts.

In the majority of cases (n = 6, 85.7%) an end-to-side anastomosis was used to preserve distal limb blood flow. In one case (n = 1, 14.3%) a flow-through anastomotic configuration was used (Table 3).

### 4.6. Steal Syndrome Occurrence and Management

In total, 7 patients developed steal syndrome. In all cases, the flap survived, but the distal blood flow to the lower extremity was compromised. Among these patients, four (n = 4, 57.1%) in the studies by Sonntag et al. [22] and Musser et al. [23] did not undergo further salvage interventions, and a below-the-knee amputation (BKA) was performed (Table 4).

In the study by Rainer et al. [24], one patient developed post-operative complications related to the microvascular anastomosis, which required revision of the venous outflow anastomosis on post-operative day 4, followed by flap debridement on post-operative day 22. Despite these interventions, progressive ischemic necrosis of the distal foot and remaining toes led the surgical team to opt for a Chopart amputation on post-operative day 22. Further debridement of peripheral necrosis was performed on post-operative days 58 and 64. In the remaining two cases from Rainer et al. [24], blood flow to the lower extremity was compromised, and amputation was performed just proximal to the flap, although the authors did not provide further details.

The follow-up period for the patients in the studies by Sonntag et al. [22] and Musser et al. [23] is not specified. However, in the study by Rainer et al. [24], the mean follow-up period for patients who developed steal syndrome was 37.7 ± 4.6 months (Table 4).

### 4.7. Risk of Bias and Level of Evidence

Case reports and case series, evaluated using the methodological quality assessment tool, demonstrated adequate documentation of outcomes, clear selection methods, and evidence suggesting causality, such as decreased blood flow to the distal extremity (steal phenomenon) as identified by Doppler ultrasound (Table 5). However, we can infer that the steal phenomenon in free flap microvascular reconstruction of the lower extremity is a very rare occurrence, with only seven patients reported. The reduced sample size of the analysis could pose a significant limitation to the generalizability and reliability of the findings.

Based on the American Society of Plastic Surgery’s levels of evidence and grading recommendations, the included studies comprised two level IV studies and one level V study (Table 1).

## 5. Discussion

This systematic review highlights the rarity of steal syndrome in free flap microvascular reconstruction of the lower extremity, while also emphasizing its significant impact when it does occur. A total of seven patients developed steal syndrome across the studies, indicating that although this complication is uncommon, it can still arise in certain cases. Steal syndrome refers to the phenomenon when blood is diverted from the native tissue to the free flap, leading to tissue necrosis in the lower limb. This occurs due to impaired blood flow, where the recipient artery supplying the flap becomes overwhelmed, resulting in insufficient perfusion to the surrounding tissues. As a result, the lower extremity becomes ischemic, which poses significant challenges to recovery and can ultimately lead to complications, including the potential loss of the limb.

The studies reviewed suggest that several clinical and anatomical characteristics may predispose patients to developing steal syndrome following free flap microvascular reconstruction. Notably, comorbidities such as hypertension, diabetes, and peripheral vascular disease (PVD) were common among all patients who developed steal syndrome. These comorbidities likely contribute to impaired vascular integrity and reduced tissue perfusion, both of which are critical factors in the development of steal syndrome. The presence of PVD in most patients across the studies further supports the notion that patients with poor baseline vascular health are more likely to develop steal syndrome. PVD is characterized by the obstruction of blood flow within the arterial tree, excluding the intracranial and coronary circulations. The primary cause of PVD in all patients is atherosclerosis, which results in the narrowing or blockage of arteries. This condition significantly impairs the body’s ability to sustain adequate blood flow, even in the context of reconstructive surgery, thereby complicating recovery and increasing the risk of tissue ischemia and necrosis [25]. Sonntag et al. explain the steal phenomenon as blood being diverted to the conduit of the least resistance (flap) [22]. This issue is compounded by the use of myocutaneous flaps (such as the LD flap), which require significant and consistent blood flow to ensure survival of both the muscle and skin components.

According to Ohm’s law [26], which is commonly used in electrical circuits, the relationship between current (A), voltage (V), and resistance (R) is given by the following formula:

(1) $$A=\frac{V}{R}$$

In fluid dynamics, Poiseuille’s law provides an equivalent relationship for flow through a conduit [27]. The law details that flow (Q) is directly proportional to perfusion pressure (P) and inversely proportional to the resistance (R). The formula for flow in Poiseuille’s law is as follows, where (Q) is flow, (P) is perfusion pressure, (r) is radius, (l) is length, and (μ) is viscosity:

(2) $$Q=\frac{P\pi r^{4}}{8l\mu }$$

In parallel circuits, the total resistance is provided by the following formula:

(3) $$\frac{1}{R_{total}}=\frac{1}{R_{1}}+\frac{1}{R_{2}}$$

As a result, flow to the overall circuit will increase in response to the lowered resistance achieved through the free flap. This is the argument behind the steal phenomenon in lower limb microvascular reconstruction.

The use of vein grafts was common in the studies, with 4/7 cases involving saphenous vein and PTFE grafts. These grafts were used to achieve continuity between stenosed/thrombosed proximal and distal segments of the recipient artery. The presence of vein grafts in the lower limb most likely increases peripheral resistance [28,29] and serves as a negative predictor of steal syndrome, exemplifying the negative role of PVD in the occurrence of the steal phenomenon. Additionally, peripheral neuropathy was observed in one case, which could further compromise vascular health and tissue recovery.

Notably, all of the flaps used in these studies (LD, rectus abdominis, and gracilis) are myocutaneous flaps, which are known to have a reliable blood supply but also require adequate blood flow to survive [30]. The majority of the anastomoses (n = 6/7, 85.7%) were performed end-to-side (ETS), with only one case (n = 1/7, 14.3%) utilizing a flow-through technique. This suggests that steal syndrome may be more prominent in cases with ETS anastomoses and in myocutaneous flaps, which require a consistent and robust blood supply. These factors may serve as negative predictors that increase the likelihood of developing steal syndrome.

Although the majority of cases of steal syndrome in our review were associated with ETS anastomoses, there is some contradiction in the literature regarding the relationship between anastomosis type and the development of steal syndrome. syndrome. However, a study by Motomiya et al. [31] evaluating blood flow distribution following ETS anastomosis with a wide arteriotomy technique in free flap extremity reconstructions found no cases of steal syndrome after evaluating 20 free flaps (18 patients). While the authors did not observe any steal phenomenon, the study also highlighted that the size of the arteriotomy did not significantly affect the blood flow volume in the flap. Furthermore, flap type was found to play a crucial role, with myocutaneous flaps exhibiting significantly higher blood flow than fasciocutaneous flaps [31]. Despite using a wide arteriotomy, the study found no compromised distal circulation, suggesting that the arteriotomy size itself does not contribute to the development of steal syndrome. This conclusion challenges the assumption that larger arteriotomies necessarily lead to vascular complications. Although this study was excluded from the review due to the absence of steal syndrome cases, its findings remain highly relevant, as they underscore that steal syndrome may not be directly influenced by arteriotomy size and that the surgical technique itself may play a critical role in determining outcomes.

Another study by Nasir et al. evaluated blood flow changes in the recipient artery, flap pedicle, and distal leg circulation following flow-through free latissimus dorsi muscle flap reconstructions [32]. Interestingly, despite the potential for increased blood flow in the recipient and pedicle arteries, the study demonstrated that flow-through flaps did not disturb distal leg circulation, even in the presence of elevated blood flow in the flap’s vascular pedicle. While this study was excluded from our analysis due to the absence of steal syndrome cases, its findings are noteworthy. Nasir et al. underscore that despite increased blood flow in the flap’s vascular pedicle, flow-through flaps did not compromise distal circulation, implying that other factors, in addition to surgical technique, may be involved in the development of steal syndrome. Thus, while ETS anastomoses were the most common in the cases with steal syndrome in our review, the studies by Motomiya et al. [31] and Nasir et al. [32] collectively suggest that steal syndrome may be multifactorial, with surgical technique being only one of the contributing elements. These contradictions in the literature highlight the complexity of steal syndrome and its underlying mechanisms.

The management of steal syndrome in free flap microvascular reconstruction, as observed in the included studies, primarily involves a combination of surgical intervention and vascular revision. In cases where distal blood flow to the lower extremity was compromised, amputation was often the final outcome, with below-the-knee amputation (BKA) being the most common procedure (n = 4, 57.1%) [22,23]. This intervention was deemed necessary when ischemic necrosis progressed despite initial efforts to salvage the limb. In some instances, flap revision and debridement were performed to address complications related to microvascular anastomosis, such as venous insufficiency. For example, in the study by Rainer et al. [24], one patient required an anastomotic revision on post-operative day 4 and further flap debridement on day 22 due to progressive ischemia. However, despite these efforts, the patient ultimately underwent a Chopart amputation due to extensive necrosis. These findings suggest that while surgical revision and debridement can offer temporary solutions, amputation remains a frequent outcome when ischemia becomes irreversible.

A notable limitation in the studies reviewed is the lack of follow-up information in some cases, particularly in the studies by Sonntag et al. [22] and Musser et al. [23], where the duration of follow-up was not specified. While this absence of long-term outcome data is a drawback, it is important to note that steal syndrome is typically a complication that occurs in the immediate post-operative period. As such, the long-term follow-up may not be as critical, as steal syndrome generally manifests early on, and its management—typically through anastomotic revision, or amputation if revision fails—addresses the immediate concerns related to the condition. In cases where amputation is performed, the complication is effectively addressed, and there is no ongoing need for extended follow-up. However, the study by Rainer et al. [24] did provide valuable insights, with a mean follow-up period of 37.7 ± 4.6 months for patients who developed steal syndrome, offering a more comprehensive understanding of the progression and resolution of this complication. While the lack of follow-up in some studies limits our understanding of long-term outcomes, the immediate post-operative nature of steal syndrome means that the impact on patient health and quality of life can be adequately assessed in the short term.

## 6. Limitations

While the studies included in this review provide valuable insights into the incidence and management of steal syndrome, they have several inherent limitations. One key limitation is the small sample size, with only seven patients across all studies, which limits the generalizability of the findings [33,34]. Additionally, the studies primarily consist of case reports and case series, which do not allow for robust statistical analyses or definitive causal inferences [35]. The absence of randomized controlled trials (RCTs) or comparison groups further restricts the ability to establish clear cause-and-effect relationships between surgical techniques, patient characteristics, and the development of steal syndrome [36,37,38].

Moreover, the studies included in this review are classified as level IV and V studies according to the American Society of Plastic Surgery’s levels of evidence [21], reflecting a moderate-to-low level of evidence. This classification emphasizes the need for more high-quality studies, including prospective trials with larger sample sizes and randomized designs, to more definitively assess the risk factors and management strategies for steal syndrome in reconstructive surgeries. Additionally, the rarity of steal syndrome may contribute to its underreporting in the literature, further highlighting the need for more comprehensive studies to better understand its incidence and clinical impact.

Despite these limitations, the methodological quality of the studies was generally adequate, with clear documentation of outcomes and well-defined selection criteria [39,40].

## 7. Conclusions

In conclusion, the findings from this systematic review suggest that steal syndrome appears to be a rare phenomenon, with limited cases described in the literature. The studies included in this review predominantly feature older patients with extensive peripheral vascular disease (PVD) and diabetes, suggesting that steal syndrome is more likely to occur in individuals with preexisting vascular issues. Given the scarcity of cases, it remains debatable whether steal syndrome should be a significant concern in younger patients with otherwise healthy vascular systems. In conclusion, the findings suggest that while steal syndrome remains uncommon, it highlights the importance of recognizing its potential impact, particularly in patients with preexisting vascular conditions. The studies indicate that compromised distal blood flow often results in significant complications, ultimately leading to amputation in the majority of cases. Future research should focus on larger-scale studies and prospective trials to better understand the anatomical and clinical predictors of steal syndrome, particularly in patients with known risk factors such as PVD and diabetes. Furthermore, detailed investigations into the role of flap type, anastomosis technique, and vascular pedicle choice in the development of steal syndrome are warranted. As current management primarily involves amputation when ischemia persists, there is a pressing need for further exploration of more effective treatment strategies for managing steal syndrome and ultimately preventing amputations. Enhanced understanding of this complication could lead to better patient selection, early intervention, and improved surgical outcomes in the future.

## Figures and Tables

**Table 1:** Basic study characteristics.

Study	Journal	Study Design	Country	Patients	Number of Flaps	Level of Evidence
**Sonntag et al., 1995** [22]	*Annals of Plastic Surgery*	USA	Retrospective case series	3	3	IV
**Musser et al., 1995** [23]	*European Journal of Plastic Surgery*	USA	Case report	1	1	V
**Rainer et al., 2003** [24]	*Journal of Reconstructive Microsurgery*	Austria	Retrospective case series	9	10	IV

**Table 2:** Clinical reporting and demographic characteristics of the studies.

Study	Mean Age (±SD)	Indication	Flap Type	Number of Patients Developing Steal Syndrome	Patient Comorbidities	Vascular Pedicle	Recipient Vessel
**Sonntag et al., 1995** [22]	69.67 ± 4.04	Non-healing heel ulcer and necrosis of the 5th toe, non-healing heel ulcer, non-healing heel ulcer, and exposed Achilles tendon	LD (2) and rectus abdominis (1)	3	HTN, DM, PVD, and RF	Thoracodorsal and inferior epigastric	Popliteal (3)
**Musser et al., 1995** [23]	73	Non-healing forefoot ulcer	LD	1	HTN, DM, and PVD	Thoracodorsal	Anterior tibial
**Rainer et al., 2003** [24]	54.3 ± 9.37	Diabetic foot ulcer	LD (2) and gracilis (1)	3	DM, HTN, PVD, and PN	Thoracodorsal, medial circumflex femoral	Anterior tibial (1) and dorsal pedis (2)

**Notes:** LD: latissimus dorsi, HTN: hypertension, DM: diabetes mellitus, PVD: peripheral vascular disease, RF: renal failure.

**Table 3:** Microvascular anastomotic characteristics of the studies.

Study	Anastomotic Configuration	Artery Inflow (Recipient Artery)	Venous Outflow (Single/Double Vein Anastomosis)	Vein Graft Used (Yes/No)
**Sonntag et al., 1995** [22]	End-to-side	Reverse vein graft between the LD and popliteal artery, vein graft used on PTFE graft between the deep femoral artery and popliteal artery, and vein graft between the popliteal artery and dorsal pedis	Popliteal vein (single vein)	Saphenous vein
**Musser et al., 1995** [23]	End-to-side	Saphenous vein graft between the superficial femoral to the proximal anterior tibial	Posterior tibial vein (single vein)	Cephalic vein
**Rainer et al., 2003** [24]	Flow-through and end-to-side	Anterior tibial	Anterior tibial (single vein)	No

**Table 4:** Clinical outcomes of the studies.

Study	Patients with Steal Syndrome	Salvage Intervention	Follow-Up (Months)
**Sonntag et al., 1995** [22]	3	Not attempted, BKA	Not mentioned
**Musser et al., 1995** [23]	1	Not attempted, BKA	Not mentioned
**Rainer et al., 2003** [24]	3	Anastomotic revision of venous thrombosis (post-op day 4), debridement (post-op day 22),	37.7 ± 4.6
		Chopart’s amputation due to progressive ischemic necrosis of the remaining toes (after post-op day 22),	
		and debridement of peripheral necrosis (post-op day 58 and 64)	

**Table 5:** A qualitative assessment of the case report and case series included.

Domain for Evaluating the Methodological Quality of Case Reports and Case Series								
	Selection		Ascertainment		Causality		Reporting	
Reference	Q.1	Q.2	Q.3	Q.4	Q.5	Q.6	Q.7	Q.8
Sonntag et al., 1995 [22]	Yes	Yes	Yes	Yes	Yes	Yes	Yes	Yes
Musser et al., 1995 [23]	Yes	Yes	Yes	Yes	Yes	Yes	Yes	Yes
Rainer et al., 2003 [24]	Yes	Yes	Yes	Yes	Yes	Yes	Yes	Yes

**Selection:** [Q.1] Does the patient(s) represent(s) the whole experience of the investigator (center) or is the selection method unclear to the extent that other patients with similar presentations may not have been reported? **Ascertainment:** [Q.2] Was the exposure adequately ascertained? [Q.3] Was the outcome adequately ascertained? **Causality:** [Q.4] Were other alternative causes that may explain the observation ruled out? [Q.5] Was there a challenge/rechallenge phenomenon? [Q.6] Was there a dose–response effect? [Q.7] Was follow-up long enough for outcomes to occur? **Reporting:** [Q.8] Is the case(s) described with sufficient detail to allow other investigators to replicate the research or to allow practitioners to make inferences related to their own practice?

## Supplementary Materials

The following supporting information can be downloaded at: https://www.mdpi.com/article/10.3390/bioengineering12060647/s1.
