# Supplementary material for: Steal Syndrome in Free Flap Microvascular Reconstruction of the Lower Extremity: Systematic Review of Incidence, Risk Factors, and Surgical Management
Source: Bioengineering (Basel). 2025 Jun 12;12(6):647. doi: 10.3390/bioengineering12060647 (PMC12189075; doi:10.3390/bioengineering12060647)
Supplement: Supplementary file 1 [file bioengineering-12-00647-s001.zip › bioengineering-3674204-supplementary.pdf]

Supplementary Materials

Search Strategy

MEDLINE (Ovid interface, 1946 – January 28, 2025) – 14 references retrieved on 29 January 2025

- **Multi-line search run in the Advanced Search interface:**

1. Free Tissue Flaps/
2. ('micro anastomosis' or 'microanastomosis' or (('micro vascular' or microvascular) adj (surger* or reconstruction* or anastomosis)) or (free adj2 (flap* or graft*))).ti,ab,kf.
3. 1 or 2
4. exp Subclavian Steal Syndrome/
5. ((steal adj (syndrome* or phenomen*)) or ((arter* or vascular or Subclavian) adj steal)).ti,ab,kf.
6. 4 or 5
7. 3 and 6
8. 7 not (Animals/ not (Animals/ and Humans/))

Embase (Elsevier interface) – 22 references retrieved on 29 January 2025

- **One-line search run in the Quick search tab of the Embase.com interface:**

1. ('artery steal syndrome'/exp OR ((steal NEXT/1 (syndrome* OR phenomen*)) OR ((arter* OR vascular OR Subclavian) NEXT/1 steal)):ti,ab,kw) AND
2. ('free tissue graft'/exp OR 'microvascular surgery'/exp OR ('micro anastomosis' OR 'microanastomosis' OR (('micro vascular' or microvascular) NEXT/1 (surger* or reconstruction* or anastomosis)) OR (free NEAR/2 (flap* OR graft*))):ti,ab,kw
3. NOT (('animal'/exp or 'nonhuman'/exp) NOT 'human'/exp)

Cochrane Central Register of Controlled Trials (WileyOnline interface) – 0 references retrieved on 29 January 2025

- **Using Search Manager in Advanced Search:**

1. [mh "free tissue flaps"] or [mh "microvascular surgery"]
2. ("micro anastomosis" OR "microanastomosis" OR (("micro vascular" or microvascular) NEXT/1 (surger* or reconstruction* or anastomosis)) OR (free NEAR/2 (flap* OR graft*))):ti,ab,kw
3. #1 or #2
4. [mh "Subclavian Steal Syndrome"]
5. ((steal NEXT/1 (syndrome* OR phenomen*)) OR ((arter* OR vascular OR Subclavian) NEXT/1 steal)):ti,ab,kw
6. #4 or #5
7. #3 and #6
8. #7 NOT ([mh "juvenile"] NOT [mh "adult"])

Scopus (Elsevier interface) – 23 references retrieved on 29 January 2025

- **One-line search run in the Advanced Search interface:**

1. TITLE-ABS-KEY (((steal PRE/0 (syndrome* OR phenomen*)) OR ((arter* OR vascular OR Subclavian) PRE/0 steal)) AND ("micro anastomosis" OR "microanastomosis" OR (("micro vascular" or microvascular) PRE/0 (surger* or reconstruction* or anastomosis)) OR (free W/1 (flap* OR graft*))))
